# Financial awareness and business development cognition positively influence the sustainable development of rural family businesses

**DOI:** 10.3389/fsoc.2025.1569713

**Published:** 2025-07-11

**Authors:** Zheng Xingpeng, Jacquline Tham, Ali Khatibi

**Affiliations:** ^1^School of Management, Chongqing Institute of Engineering, Chongqing, China; ^2^Postgraduate Centre, Management and Science University, Shah Alam, Malaysia

**Keywords:** family business operators, business development cognition, financial awareness, abilities to run businesses, sustainable development of family businesses

## Abstract

Family businesses are the primary form of economic organization worldwide. In most countries, family businesses account for 70 to 90% of the annual global gross domestic product (GDP) and 50 to 80% of all jobs. In rural areas of China, the number of family farms engaged in cultivation is estimated to be up to 4 million as of October 2023. Family businesses exhibit unique characteristics compared to non-family businesses, and further research is needed on how to promote their sustainable development. Therefore, we constructed structural equation model (SEM) to explore the role of family business operators’ business development cognition and financial awareness in sustainable development and investigated 507 family business operators of rural cultivation. We used SPSS 25.0, AMOS 24.0 to analyse the data. Research results indicated that the business development cognition (β = 0.327, *p* < 0.001) and financial awareness of family business operators (β = 0.294, *p* < 0.001) positively influenced the sustainable development of family businesses. In this process, the abilities of family business operators to run businesses played a moderating role. This study complemented and improved the Upper Echelons Theory (UET), and pointed out that the ability of CEOs alone was not enough to promote the development of rural family firms, and the combination of CEO’s cognition of business development, financial awareness and abilities to run businesses was needed to promote the sustainable development of rural family firms. Then, several implications have been provided for family business operators.

## 1 Introduction

Public data from the Ministry of Agriculture and Rural Affairs of China showed that as of October 2023, nearly 4 million family farms were included in China’s family farm directory associations. In most countries, family businesses account for 70 to 90% of the annual global gross domestic product (GDP) and 50 to 80% of all jobs (Firfiray and Gomez-Mejia, 2021). Family businesses are the primary form of economic organization worldwide (Ratten, 2023).

However, the development model of family businesses differs from that of other economic organizations (Chrisman et al., 2003). Family businesses exhibit unique characteristics compared to non-family businesses, and more research is needed on how to promote their growth and sustainability (Soares et al., 2021). Family business operators, especially in rural areas, often discontinued their family business midway, resulting in the family business not surviving for a long time (Zhang and Zhang, 2021). For this situation, the studies reviewed in the field of family business research from 2012 to 2022 also pointed out that individual behavior in family businesses had not been explored in depth so far. Future researchers can explore the continuity of business development from the perspective of individual behavior in family businesses (Siaba and Rivera, 2024). Therefore, the study of the influencing factors on the sustainable development of rural family businesses is significant.

Several key issues are the role of human capital in influencing the sustainable development of family businesses. Well-educated employees add to the company’s knowledge wealth through their daily performance and accelerate external knowledge linkages by interacting with individuals outside the company who have relevant competencies (Lund Vinding, 2006). Not only did the human capital of the operator, such as knowledge, skills, and abilities, have an impact on the development of the family business, but also the attitudes and motivations of the family business operator, which also had an impact on the sustainable development of the family business (Dawson, 2012). Entrepreneurs’ human capital, including age, level of higher education, and experience, facilitated access to business opportunities and thus had a positive impact on firm performance (Choi and Chang, 2020). The human capital of family business operators is crucial in managing family firms in India, as it helps balance familial relationships with business pragmatism to ensure sustainable development (Rajan et al., 2020). A key factor influencing the survival of family firms is their ability to adapt and cope with existing and pressing challenges (Duarte Alonso et al., 2023). The entrepreneurial competencies of family business managers helped them to seize entrepreneurial opportunities, realize business value propositions, and drive sustainable business growth (Jia and Hou, 2024). In business adjustment strategies, integrating the organization’s experience with current realities promotes the sustainable development of the company (Istipliler et al., 2024). Leadership, motivation, qualifications, satisfaction, and creativity significantly impact the sustainable development of family businesses, influencing the external dimensions of a firm’s surviving capital (Więcek-Janka et al., 2024). The educational background and expertise of managers significantly impacted earnings management within Indonesian banks, as the diversity of educational backgrounds and experiences enhanced management and made the organization more efficient (Aryani et al., 2024). The management team’s big data analytic abilities enabled organizations to effectively monitor and anticipate changes in customer behavior, competitor behavior, and market demand, thereby positively influencing the organization’s strategic outcome (Achyar, 2024). Family business leaders must recognize that artificial intelligence (AI) empowers a highly skilled workforce and boosts their net earnings, which can be highly beneficial for the growth of a family business (Birkbeck and Rowe, 2024). A high level of human capital capacity implied a greater capacity to learn, which in turn improved the firm’s ability to innovate and grow (Baiyegunhi, 2024). In conclusion, current researchers have discussed the roles of human capital in the sustainable development of family businesses.

Notably, the factors affecting the sustainability of family businesses appear to be diverse. In addition to human capital, a few researchers have noted other factors. Kumar and Kumar (2023) argued that the financial awareness of entrepreneurs contributes to their financial literacy, which in turn affects their business performance through the combined effect of financial inclusion. Financial awareness among entrepreneurs can help SMEs better access finance and avoid financial risks, promoting sustainable business growth (Ye and Kulathunga, 2019). Good financial awareness among entrepreneurs can help them gain a competitive advantage, resulting in a positive and profitable business (Yakob et al., 2021). Entrepreneurial financial awareness can significantly influence the adoption of bank credit operations by SMEs, thereby impacting the business performance of these enterprises (Bawono et al., 2022). It is crucial to scientifically cultivate the financial awareness of small entrepreneurs, as this can enhance the credit risk control ability of small enterprises (Gu et al., 2024). Financial awareness among entrepreneurs plays a positive role in enterprise development, and its impact on rural family businesses can be further explored.

In addition to financial awareness, some scholars have recognized the important role of individual cognition. They conducted a qualitative analysis only and did not delve into empirical arguments (Sharma et al., 2020). In innovation decision-making, new venture executives analyze the environment and identify innovation opportunities based on unique cognition of business development, which is an important aspect that influences firm development (Zhou et al., 2021). In an ever-changing environment, the business development cognition of executives helped firms to accurately grasp customer needs, analyze market trends, and drive firm growth (Narayan et al., 2021). Entrepreneurs’ business development cognition enhanced the entrepreneurial patchwork, which in turn influenced firm innovation and development (Hou et al., 2022). Similarly, the business development cognition of entrepreneurs has been shown to be instrumental in the development of firms. How well it performs in family firms is also a meaningful issue that can be further argued.

Additionally, the business abilities of business operators were also a topic that was mentioned. The ability of entrepreneurs to run businesses (managerial and marketing abilities) moderated the relationship between the competitive strategies of Ghana’s small firms (cost leadership and differentiation) and the performance of the firm’s development (Acquaah and Agyapong, 2015). The operational competence at the top of the organization is crucial for the firm’s development and is closely tied to its understanding of the policy and dynamic environment (Von den Driesch et al., 2015). It has been argued that managers with strong business operations skills could better predict future earnings and effectively manage future cash flows to generate higher profitability (Libby and Rennekamp, 2016). In addition, the CEO’s ability to run businesses had a significant impact on the firm’s financial resources, debt, equity, and capital structure (Matemilola et al., 2018). Top executives with operational business experience had access to scarce resources and strengthened the relationship between intellectual capital (education and cognition) and resource acquisition, which can be highly beneficial for business growth (Ying et al., 2019). The abilities of entrepreneurs to run businesses (including digital literacy, experience, and financial literacy) enabled better operation of the business and contributed to the sustained growth of SMEs with the moderating effect of resource acquisition (Sualeh Khattak et al., 2024). Inspired by this, the study combines social cognitive theory (Bandura, 1999) and upper echelons theory (Hambrick and Mason, 1984) and focuses on how the rural family business operators’ business development cognition, financial awareness, and abilities to run businesses can influence the sustainable development of their family businesses. 2 research questions are proposed.

*RQ1*: How do rural family business operators’ business development cognition and financial awareness affect the sustainable development of family businesses?

*RQ2*: What roles do rural family business operators’ abilities to run businesses play in the sustainable development of family businesses?

## 2 Literature review and research hypotheses

### 2.1 Social cognitive theory and the sustainable development of rural family businesses

Bandura proposed a social cognitive theory, which is based on social learning theory. Social cognition theory suggests that people are subjective and self-regulating. They not only passively reflect the social environment but also actively shape and change it (Liñán and Fayolle, 2015). By applying social cognitive theory, scholars have discussed the role of management cognition in business development. Management cognition refers to the beliefs or mental models held by strategic decision-makers in a firm that reflect the state of the external environment, strategy, business portfolio, or organization (Porac and Thomas, 2002). The interaction between development cognition and organizational positioning had a significant impact on the renewal of corporate strategy (Eggers and Kaplan, 2009). Cognitive errors of managers can lead to the rigidity of a firm’s core competencies, thus hindering its sustainable development (Guo et al., 2022). Business development, cognition, and financial awareness are two types of individual cognition. Drawing on these results, the study proposes that business development cognition and financial awareness play key roles in the sustainable development of rural family businesses.

### 2.2 Upper echelons theory and the sustainable development of rural family businesses

The upper echelons theory (UET) suggested that the characteristics and behaviors of an organization’s managers could have a significant impact on the organization’s strategic choices and performance, emphasizing the role of factors such as the demographic characteristics, values, cognition, and abilities of the management team in organizational decision-making and development (Hambrick and Mason, 1984). While using UET, many researchers also argued that factors influence business development. The cognition shaped by managers’ psychological, sociological, and experiential attributes strongly influenced the behaviors of managers running businesses (Hambrick, 2007). The ability of family business leaders to run businesses is a key source of organizational culture, fostering internal integration among members and creating the conditions for the sustainable development of the family business (Efferin and Hartono, 2015). By applying UTE theory, Quinn et al. (2024) found that Nigerian brewery managers’ abilities, including international experience, political affiliation, educational background, and professional experience, had a positive impact on the firm’s strategic choices. Through UET theory, Recendes et al. (2024) found that the abilities of top management teams influenced the relationship between the firm’s shareholders and the organization’s output outcomes. According to the UET theory, Wagdi and Fathi (2024) found that the diversity of education and foreign nationalities in the top management team attracted talent at various levels to join the organization and had a significant positive impact on business performance. In summary, entrepreneurs’ abilities to run businesses are crucial to the sustainable development of businesses. Accordingly, the study proposes that the abilities of family business operators to run businesses affect the sustainable development of rural family businesses.

### 2.3 Cognition of business development (C)

The role of cognition in the process of business sustainability has been mentioned by many researchers in other studies. Outdated cognitive processes and structures will lead to inadequate business management decisions, thus hindering the growth of the family business (Beck and Wiersema, 2013). Entrepreneurs with higher cognition of development scores tended to be more likely to engage in creative activities (Kiss and Barr, 2017). The development model enhanced cognition of human and social capital, ensuring that the firm’s value proposition, creation, and value capture mechanisms were aligned with the changing environment for sustainable development (Heubeck and Meckl, 2022). The risk to the sustainable development of rural farm businesses was that family business operators believed they could only start the appropriate planting business in conjunction with state financial subsidies, rather than focusing on market needs (Jing and Ye, 2024). Entrepreneurs’ business development cognition positively and significantly impacted the continued growth of the new firm, as cognitively flexible managers were more likely to consider an idea from different standpoints, devise numerous solutions to solve the problem, and effectively adjust their behavior across situations to select the most effective solution (Wang et al., 2023). Therefore, this study proposes Hypothesis 1.

*H1*: Rural family business operators’ business development cognition has a positive influence on the sustainable development of family businesses.

### 2.4 Financial awareness (FA)

In the process of arguing for other factors of firm development, many researchers have found a role for financial awareness in the sustainable development of firms, but further argumentation was lacking. Financial awareness is a crucial aspect and represents an interesting issue for further development, as it has a significant impact on the welfare of everyone in the future (Nga et al., 2010). Relevant government departments should establish a financial service system tailored to the needs of rural family businesses, in line with cultivating financial awareness among rural family business operators. This will enable rural family businesses to become more adept at utilizing financial tools to achieve sustainable development (Wei, 2021). In granting loans, the high level of financial awareness among family business operators enhanced their credibility, thereby speeding up the bank’s approval process and effectively improving the efficiency of financing (Tian et al., 2022). The financial literacy of the CEO of a family firm can effectively meet the family firm’s financing needs, enabling savings for its growth (Diéguez-Soto et al., 2022). In recent years, the financial awareness of family business operators in China’s Shandong Province has provided strong support for the modernization of agriculture (Bado et al., 2023). Thus, hypothesis 2 is proposed.

*H2*: The financial awareness of rural family business operators positively influences the sustainable development of family businesses.

### 2.5 Abilities to run businesses (A)

A CEO’s management abilities determine the ability to configure and deploy a company’s portfolio of assets and have become a critical success factor for goal-oriented business success (Adner and Helfat, 2003). Under the reinforcing effect of business operations management abilities, entrepreneurs with a good level of business development cognition facilitated the concrete implementation of solutions generated by better allocating resources between exploitative and exploratory innovations, contributing to the sustained growth of family businesses (Chirico et al., 2011). As the entrepreneur’s management skills in running businesses shape organizational finance decisions, the strength of management skills is a key driver of business success (Teece, 2018). Business management abilities play a moderating role in influencing business performance outcomes cognitively, as effective management skills can better allocate resources and lead to the implementation of solutions when entrepreneurs face complex business environments (Wang et al., 2023). The ability of the family business leader significantly influenced the organizational culture, internal leadership, and customer relationships within the family business, which was critical to its sustainable development (Jamil et al., 2024). Therefore, the study concludes that the abilities of rural family business operators to run businesses have a moderating effect. The following hypotheses are proposed.

*H3*: Rural family business operators’ abilities to run businesses moderate the relationship between their business development cognition and the sustainable development of family businesses.

*H4*: The ability of rural family business operators to run businesses moderates the relationship between their financial awareness and the sustainable development of family businesses.

## 3 Methodology

### 3.1 Measurement constructs

**Figure 1:**
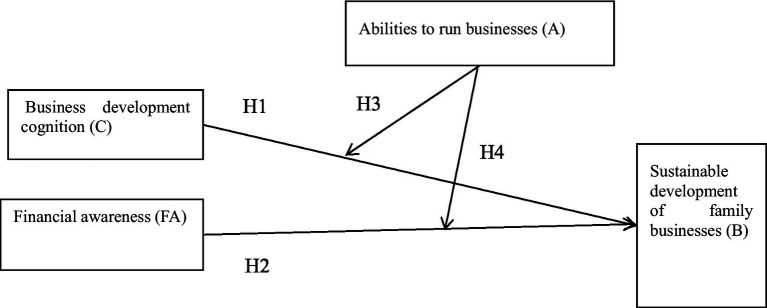
Conceptual framework.

Through the above literature collection, collation, and theoretical research derivation, the conceptual research model of this paper is presented in Figure 1. Business development cognition (C) refers to the awareness that focuses on customers’ demands, sales channels, partners, and supply chain situations, which can aid business development. Financial awareness (FA) means that family businesses cannot rely solely on their own capital and need the assistance of financial instruments to grow. Ability to run a business (A) refers to rural family business operators’ abilities to motivate employees, organize resources, and maximize the use of resources to help the business develop. The sustainable development of rural family businesses (B) ensures that the target market, talent, sales channels, profits, and other key dimensions of the rural family business can guarantee its continued growth and development.

### 3.2 Instruments

The measurements of constructs were adapted from the existing literature. The items of each construct were measured by a 5-point Likert scale, with values ranging from “1 = Strongly Disagree” to “5 = Strongly Agree.” Considering the validity of the adapted items, the measurements were sent to five experts in the field before the actual survey was conducted to solicit their evaluation of their appropriateness and advice for improvement. The measurements were revised several times following the expert’s advice and pretests, and then the new scales were adapted to suit the current study.

The construct of business development cognition comprises five measurements, originally proposed by Schrauder et al. (2018) with no changes.

The construct of financial awareness has five measurements, which are adapted from Liu et al. (2020). Such as, ‘I believe that the capital required for industrial operations should be based on own savings and should not be borrowed’ is changed to ‘I believe that the capital required for family business development should be based on own savings and should not be borrowed’, ‘I believe that a bad credit history affects the financing loan process for business development’ is changed to “I believe that a bad credit history affects the financing loan process for family business development’.

The construct of rural family business operators’ abilities to run businesses has five measurements, which are original from Guo et al. (2013) and remain unchanged.

The sustainable development of family businesses has six measurements, which are original to Zhao (2023) and remain unchanged.

### 3.3 Participants and sample

The research participants are rural cultivation operators in China whose businesses are usually founded by 2–3 family members who own all the equity and whose employees consist of self-employed people and other family members engaged in the cultivation of crops, such as planting vegetables, oranges, peaches, apples, etc., which typically belong to the family business (Sharma, 2004).

The survey commenced in the first quarter of 2024 and concluded in the second quarter of 2024, spanning approximately 6 months. The data for the study were collected through an online questionnaire. The research project team shared the study’s link to the questionnaire through the WeChat group of relevant government administrations in China, which included many rural cultivation entrepreneurs from each province. Some rural entrepreneurs received the link and voluntarily participated in the survey. It is essential to note that the valid questionnaires were completed by rural entrepreneurs who had operated their businesses in rural areas for a sufficient period, ensuring the accuracy of the key value information. Two sections of this online survey were created to examine the following influencing factors. One section consisted of questions aimed at obtaining personal information from the participants, and the other section was used to measure the hypothetical model established in the conceptual model. The questionnaire was translated into Chinese for rural entrepreneurs to complete, allowing them sufficient time to complete it in their own language.

Finally, more than 900 questionnaires were distributed to family business operators in rural areas of China, and 679 questionnaires were returned. Firstly, the study excluded 31 questionnaires that contained the same answers to multiple consecutive questions. Secondly, 14 questionnaires with missing values were removed. Thirdly, the study deleted 127 questionnaires that took a short time (the mean minus more than 2 standard deviations) and a long time (the mean plus more than 2 standard deviations). Finally, the study utilized a total of 507 valid samples, representing 74.6% of the recovery rate. The sample size is adequate (Bougie and Sekaran, 2019).

### 3.4 Data analysis techniques

SPSS 25.0 and AMOS 24.0 were used to analyze the data in this study (Do-Thi and Do, 2022; Ramadani et al., 2022). In the first step, the descriptive statistics were used to determine the internal consistency of constructs by calculating Cronbach’s alpha (*α*) (shown in Table 1). In the second step, the confirmatory factor analysis (CFA) was used to assess the model fit and structural validity. Third, Harman’s single-factor test was used to evaluate common method bias. Finally, the hypothesis test and moderating effects of abilities to run businesses were tested using AMOS 24.0 and the SPSS PROCESS technique (Khan et al., 2024), which are analyzed in the later section.

**Table 1:** Analysis of reliability and validity for variables.

Constructs	Items	Loadings	α	CR	AVE
Sustainable development of family businesses	B1	0.771	0.896	0.897	0.591
	B2	0.755			
	B3	0.779			
	B4	0.776			
	B5	0.754			
	B6	0.776			
Business development cognition	C1	0.798	0.889	0.889	0.617
	C2	0.773			
	C3	0.786			
	C4	0.781			
	C5	0.788			
Financial awareness	FA1	0.788	0.888	0.888	0.614
	FA2	0.792			
	FA3	0.773			
	FA4	0.787			
	FA5	0.777			
Abilities to run businesses	A1	0.811	0.901	0.901	0.646
	A2	0.793			
	A3	0.791			
	A4	0.804			
	A5	0.820			

α, Cronbach’s alpha; AVE, average variance extracted; CR, construct reliability.

## 4 Results

### 4.1 Demographic profile

The demographic characteristics of the respondents are shown in Table 2. Respondents originated from 25 provinces in China, with a regional distribution as shown in Table 2. The sample consisted of 52.5% male respondents (*n* = 266) and 47.5% female respondents (*n* = 241). By age, the largest age group of respondents is 50–59 years old, accounting for 53.1% (*n* = 269), followed by the 40–49-year-old group, accounting for 23.7% (*n* = 120), and the third largest group is those over 60 years old, accounting for 14.6% (*n* = 74). In terms of respondents’ positions, family business owners comprised 60% (*n* = 304), while top managers accounted for 40% (*n* = 203). Overall, the data indicate that the respondents are family business operators in rural areas, and the age and regional distribution are in line with the actual situation in these regions.

**Table 2:** The distribution of participants.

Attributes	Distribution	Frequency	Percentage	Attributes	Distribution	Frequency	Percentage
Areas	Jiangsu	34	6.7%	Areas	Ningxia	6	1.2%
	Guangdong	31	6.1%		Qinghai	2	0.4%
	Zhejiang	31	6.1%		Xizang	2	0.4%
	Shandong	30	5.9%	Gender	Male	266	52.5%
	Hubei	30	5.9%		Female	241	47.5%
	Henan	28	5.5%	Age	18–29	15	3%
	Sichuan	27	5.3%		30–39	29	5.6%
	Fujian	26	5.1%		40–49	120	23.7%
	Hunan	26	5.1%		50–59	269	53.1%
	Anhui	24	4.7%		Over 60	74	14.6%
	Hebei	23	4.5%	Position	Owner	304	60.0%
	Shanxi	22	4.3%		Top manager	203	40.0%
	Jiangxi	22	4.3%	Employees	Below 10	239	47.1%
	Chongqing	20	3.9%		10–29	126	24.9%
	Liaoning	20	3.9%		30–49	75	14.8%
	Yunnan	20	3.9%		More than 50	67	13.2%
	Guangxi	20	3.9%	Industry	cultivation	507	100%
	Shanxi	18	3.6%	Annual turnover (CNY)	Less than 3 million	231	45.6%
	Guizhou	15	3.0%		3–5 million	118	23.3%
	Xinjiang	11	2.2%		6–8 million	45	8.9%
	Hainan	11	2.2%		9–11 million	42	8.2%
	Xizang	8	1.6%		More than 11 million, less than 50 million	71	14%

### 4.2 Measurement model

#### 4.2.1 Construct validity and reliability

The construct of business development cognition consisted of five measurements. The Cronbach’s alpha for the construct was 0.889. The construct’s chi-square/df was 2.341, which is less than 5. The CFI is 0.995, which is more than 0.8. RMSEA was 0.051, which is less than 0.08. The CFA results indicated excellent structural validity.

The construct of financial awareness consisted of five measurements. The Cronbach’s alpha for the construct was 0.888. The construct’s chi-square/df was 1.165, which is less than 5. The CFI is 0.999, which is more than 0.8. RMSEA was 0.018, which was less than 0.08. The CFA results indicated excellent structural validity.

The construct of abilities to run businesses consisted of five measurements. The Cronbach’s alpha for the construct was 0.901. The construct’s chi-square/df ratio was 2.087, which is less than 5. The CFI was 0.996, which is more than 0.8. RMSEA was 0.046, which is less than 0.08. The CFA results indicated excellent structural validity.

The sustainable development of family businesses consisted of six key measurements. Cronbach’s alpha was 0.896. The Cronbach’s alpha for the construct was 0.896. The CFA results indicated excellent structural validity: the CHI square/df ratio was 2.062, which was less than 5; the CFI was 0.994, which exceeded 0.8; and the RMSEA was 0.046, which was less than 0.08. The CFA results indicated excellent structural validity.

All constructs’ Cronbach’s alpha values were above 0.7, and the CR of all constructs was more than 0.7, indicating good reliability. The AVE values of all constructs were above 0.5, and the factor loadings for all constructs were greater than 0.6, indicating good validity (Hair et al., 2021). This is shown in Table 1.

#### 4.2.2 Discriminant validity

After analyzing structural validity, this study also used the Fornell-Larcker Criterion to assess the discriminant validity of each construct (Table 3). The Fornell-Larcker Criterion for constructs shows that AVE’s square root (in bold) is more than the correlations of each construct, which indicates that discriminant validity was met (Hair et al., 2019).

**Table 3:** Fornell-Larcker criterion of constructs.

Constructs	Cognition of business development	Financial awareness	Sustainable development of family businesses
Cognition of business development	**0.785**		
Financial awareness	0.496	**0.783**	
Sustainable development of family businesses	0.473	0.456	**0.768**

The bold section is AVE’s square root.

Then, Harman’s single-factor test was used to evaluate common method bias. Since the data were from a single source, they were collected through the self-reports of the questionnaire participants. This led to common method variance (Lindell and Whitney, 2001). The common method covariance between predictor and criterion variables was due to the same data source, the same measurement environment, and the characteristics of the items themselves, which were known as common method bias (Chin et al., 2012). The common method variance and common method bias mean much the same thing, but the difference is that the concept of common method variance is used to objectively describe the magnitude of this variation, whereas the concept of common method bias attempts to establish a numerical boundary for determining how large the common method variation is and whether or not it will significantly affect the validity of the findings of the study (Deng et al., 2018). To examine common method bias, the study used Harman’s single-factor test. The test revealed that the unrotated first factor could only explain 31.4% of the total variation, which met the requirements (less than 40%) (Podsakoff et al., 2003).

### 4.3 Structural model

The goodness of fit of the model can be measured by CMIN/df (the value of the degree of freedom), RMSEA (root mean square error of approximation), IFI (incremental fit index), NFI (Normed Fit Index), and CFI (comparative fit index). A value of CMIN/df less than 5 is acceptable, an RMSEA value less than 0.08 is acceptable, and values of IFI, NFI, and CFI greater than 0.8 indicate a good fit (Anderson and Gerbing, 1988). The model’s goodness of fit was shown in Table 4.

**Table 4:** Goodness-of-fit indices for research models.

Model	CMIN/df	GFI	CFI	NFI	IFI	RMSEA
Research mode	1.231	0.970	0.995	0.973	0.995	0.021
Qualified request	<5	>0.8	>0.8	>0.8	>0.8	<0.08

**Figure 2:**
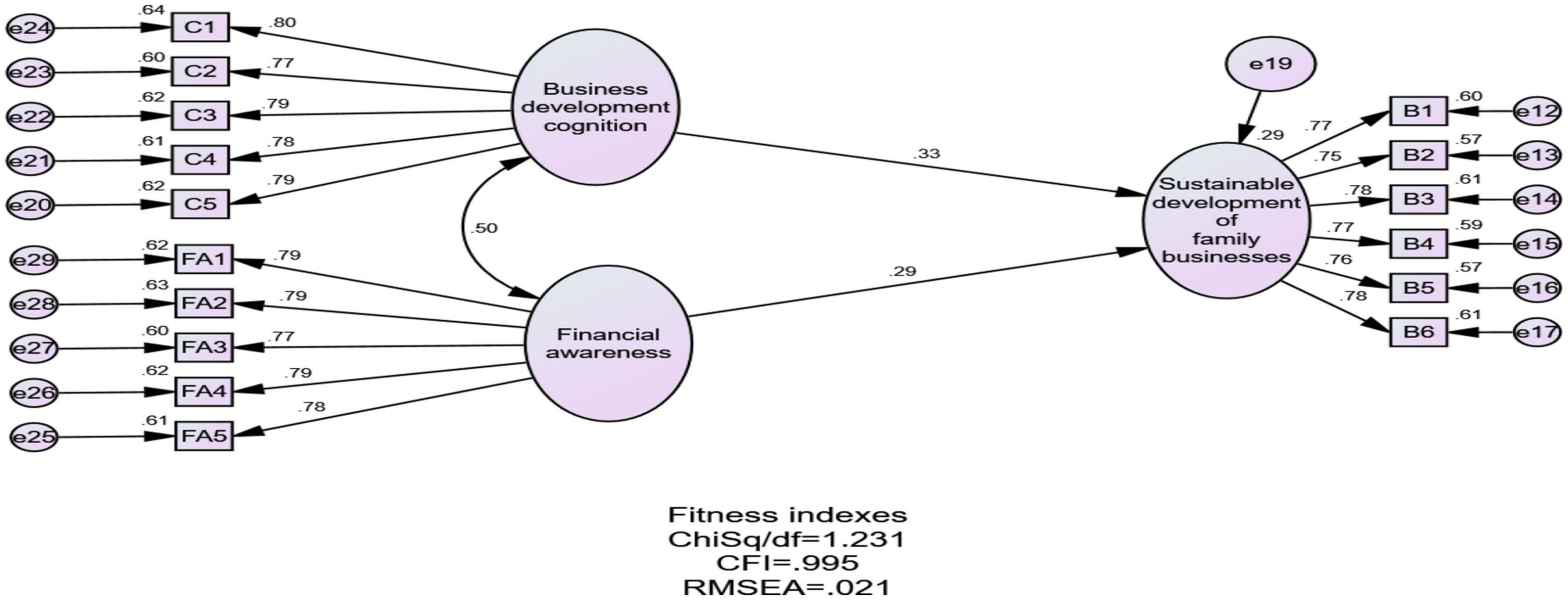
Conceptual model’s result.

The fit indices assessment of the structural equation modeling of constructs was very good (Figure 2). According to Cohen (2013) and Muniandy et al. (2021), an R^2^ of 0.26 or more indicates that the independent variable has a high predictive value for the dependent variable. The R^2^ value of the model in the study is 0.29, which is greater than 0.26. This indicates that the research model has a good predictive effect. Meanwhile, the Q^2^ value is greater than zero, and the path model is associated with some constructs (Hair et al., 2021). The Q^2^ value of the study is 0.28, which is greater than zero, indicating the predictive relevance of the variables (business development cognition and financial awareness) on the sustainable development of family businesses.

### 4.4 Hypotheses testing

#### 4.4.1 Direct effect

The cognitive understanding of business development among rural family business operators was found to positively influence the sustainable development of family businesses (*β* = 0.327, *p* < 0.001). Hypothesis H1 was supported. Financial awareness among rural family business operators was found to have a positive influence on the sustainable development of family businesses (β = 0.294, *p* < 0.001). Hypothesis H2 was supported (shown in Table 5).

**Table 5:** Results of hypothesis 1–2 testing.

Hypotheses	Hypothesized path	β	S. E.	*p*	Result
H1	Business development cognition → Sustainable development of family businesses	0.327	0.051	***	Supported
H2	Financial awareness → Sustainable development of family businesses	0.294	0.053	***	Supported

^***^p < 0.001.

#### 4.4.2 The moderating test

In Model 1, the interaction between rural family business operators’ business development cognition and abilities to run businesses was significant (β = 0.128, *p* = 0.001). Therefore, Hypothesis H3 was supported. In Model 2, the interaction between rural family business operators’ financial awareness and their ability to run businesses was significant (β = 0.18, *p* < 0.001). Therefore, Hypothesis H4 was supported (shown in Table 6).

**Table 6:** Moderating effects.

Tests	R^2^	F	t	β	*p*
Model 1 summary	0.264	60.173	-	-	***
Effect of cognition of business development on sustainable development of family businesses	-	-	6.486	0.262	***
Effect of abilities to run businesses on sustainable development of family businesses	-	-	6.305	0.247	***
Effect of Int_1 on sustainable development of family businesses	0.017	11.409	3.378	0.128	0.001
Model 2 summary	0.270	62.029	-	-	***
Effect of financial awareness on sustainable development of family businesses	-	-	6.080	0.248	***
Effect of abilities to run businesses on sustainable development of family businesses	-	-	6.091	0.241	***
Effect of Int_2 on sustainable development of family businesses	0.036	25.147	5.015	0.180	***

Int_1, cognition of business development * abilities to run businesses; Int_2, financial awareness * abilities to run businesses; ^***^p < 0.001.

**Figure 3:**
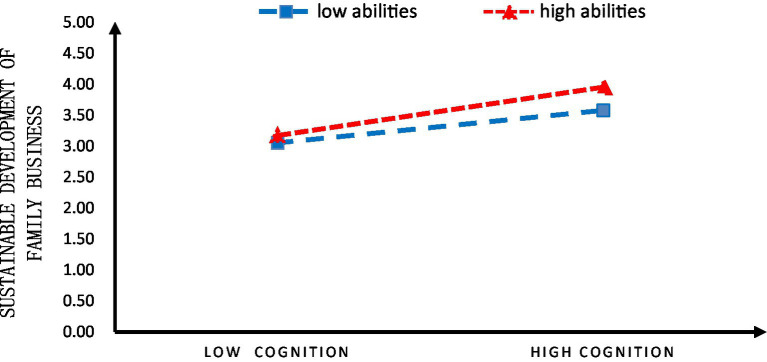
The slope of Model 1.

The rural family business operators’ abilities to run businesses played a moderating role in their business development cognition, contributing to the sustainable development of family businesses. To further understand the moderating role, the abilities of rural family business operators to run businesses were grouped as high and low, based on whether they fell above or below one standard deviation. It found that rural family business operators’ abilities to run businesses reinforced the influence of their business development cognition on the sustainable development of family businesses (Figure 3).

**Figure 4:**
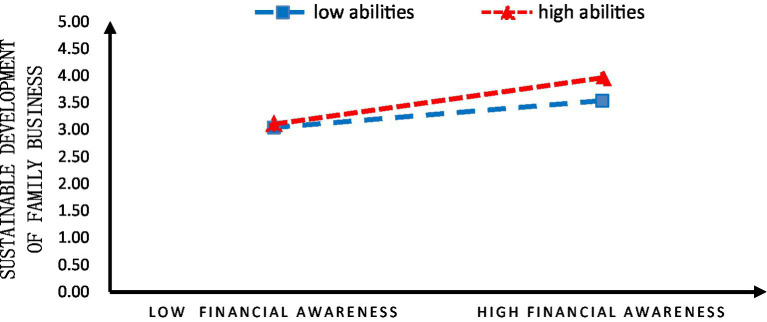
The slope of Model 2.

The rural family business operators’ abilities to run businesses played a moderating role in their financial awareness and the sustainable development of family businesses. To further understand the moderating role, the abilities of rural family business operators to run businesses were grouped as high and low, based on whether they fell above or below one standard deviation. It found that rural family business operators’ abilities to run businesses reinforced the influence of their financial awareness on the sustainable development of family businesses (Figure 4).

The results of the reconciliation test are shown in Table 7.

**Table 7:** Hypotheses 3–4 results.

Hypotheses	Hypothesized path	β	*p*	Result
H3	Rural family business operators’ abilities to run businesses moderate the relationship between their business development cognition and the sustainable development of family businesses.	0.128	0.001	Supported
H4	Rural family business operators’ abilities to run businesses moderate the relationship between their financial awareness and the sustainable development of family businesses.	0.180	***	Supported

^***^p < 0.001.

**Figure 5:**
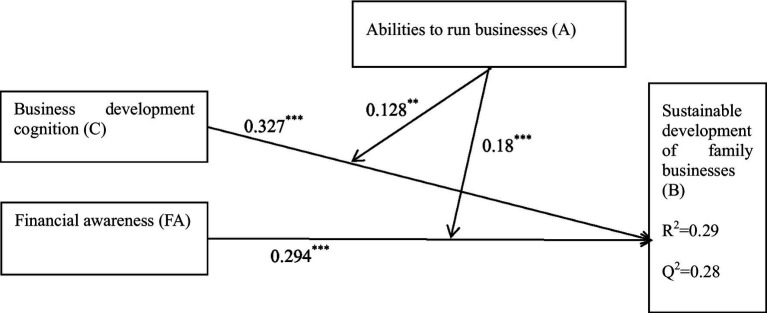
Structural equation model results with beta measure and significance. ^***^p < 0.001; ^**^p < 0.01.

The relationships among all constructs are shown in Figure 5.

## 5 Discussion

### 5.1 Finding

The study found that rural family business operators’ business development cognition and financial awareness had a positive effect on the sustained development of family businesses. The explanation is that the personal business development cognition of family business founders can influence the process of professionalization, succession, and innovation, which are key factors in the continuity of a family business (Zapata-Cantu et al., 2023). Financial awareness among rural entrepreneurs was a crucial factor in the sustainable development of their businesses (Rahman et al., 2023). The financial awareness of family business operators often helped them to make the right financing decisions to successfully achieve control of the business and pass it to the next generation, which was key to the sustainability of the family business (Jansen et al., 2023). These supported the empirical findings of H1 and H2.

Meanwhile, in the process, their abilities to run businesses played a moderating role, which strengthened the effect of their business development cognition and financial awareness on the sustained development of family firms. The reasons were that entrepreneurs’ operational abilities could modify entrepreneurial perceptions and were sources of sustained competitive advantage for firms (Teece, 2007). The entrepreneurial competence of SME entrepreneurs enabled firms to consolidate assets and resources, thereby generating new resource allocations and strengthening internal variables (managers’ cognition and motivations) to achieve sustained business growth (Ambrosini and Bowman, 2009). The growth and long-term survival of family businesses depend on the operators’ abilities to build functional business models that effectively capture and redistribute value to meet the challenges of the current business environment (Carayannis et al., 2014). The financial awareness of small business owners allowed them to recognize the importance of investing their company’s capital, while their operational capacity reinforced the scientific nature of their investment behavior (Babajide et al., 2023). These explained the empirical findings of H3 and H4.

Combining the results of H1, rural family business operators need to enhance their learning and reading skills to improve their business development cognition through platforms such as the Agricultural Science and Technology Network Bookstore, WeChat Reading, and Douban Reading. The reading content can cover business, management, history, philosophy, and other areas to broaden the boundaries of cognition. Additionally, rural family business operators must stay informed about current business reports, cutting-edge research results, and the latest business trends and developments. Industry summits, forums, and exchanges with peers and experts are ways to gain new perspectives. In addition, joining industry associations and entrepreneurs’ clubs, exchanging and interacting with outstanding entrepreneurs, and learning from their experiences is critical. Accepting feedback from employees, objectively recognizing deficiencies, engaging in self-reflection, and updating one’s self-schema are also useful practices for rural family business operators to enhance their business development cognition.

Based on the results of H2, rural family business operators focus on learning financial knowledge. Courses offered by universities, financial institutions, or the learning resources of various Internet financial platforms are all ways to learn relevant financial knowledge. Using the big AI models of the financial industry as a family business financial consultant is also a way to improve family business operators’ financial awareness.

Based on the results of H3 and H4, rural family business operators can learn advanced management concepts and methods, such as digital management and smart management, and attend relevant training courses to enhance their business management skills. In addition, regular review of the reasons for success and failure in business operations, continuous learning from experience, and summarizing shortcomings can also enhance the family business operators’ abilities to run businesses effectively.

### 5.2 Contributions and implications

In existing research, social cognitive theory is usually applied in the fields of sociology and educational research and is rarely mentioned in the field of business development (Liñán and Fayolle, 2015). Zaman et al. (2024), in their study on the impact of self-efficacy and subjective norms on entrepreneurial intentions in family businesses, also noted that there are fewer studies examining how CEOs’ cognition influences the development behavior of family firms. The study explored the impact of rural family business operators’ business development cognition and financial awareness on the sustainable development of family businesses. Of course, the study also corrected the errors of most researchers who use UET theory to study family business development by focusing only on the CEO’s human capital. This study argued that the CEO’s abilities to run businesses needed to be combined with the CEO’s business development cognition and financial awareness to better promote the sustainable development of family businesses.

The study also provided useful implications for family business operators. To maintain the sustainable development of the family business, family business operators must not forget to enhance their own business development cognition, financial awareness, and ability to run businesses while managing the family business. It also provided some warnings for operators who focus solely on the business’s profit.

### 5.3 Limitations and suggestions for future research

Due to space limitations and the requirements of the research project, the study focused on rural family business operators engaged in the cultivation industry. Industry and geographical limitations may also be limitations of the study. The cognition of family business operators in urban areas may differ from that of those in rural areas. The cognition of family business operators in other industries may also differ from that of those in the cultivation industry. Future researchers can investigate the relationship between the cognition of family business operators and the sustainability of the family business in a broader context or across multiple industries.

## 6 Conclusion

The study developed a research framework examining how rural family business operators’ business development cognition, financial awareness, and ability to run businesses impact the sustainable development of family businesses, based on social cognitive theory and Upper Echelons theory. It posed two research questions. Research question 1 is: How do rural family business operators’ business development cognition and financial awareness affect the sustainable development of family businesses? Research question 2 is: What roles do rural family business operators’ abilities to run businesses play in the sustainable development of family businesses? The results of H1 and H2 answered question 1. For every 1% increase in rural family business operators’ business development cognition, the sustainability of the family business increased by 0.327%. For every 1% increase in the financial awareness of rural family business operators, the sustainability of the family business increased by 0.294%. The results of H3 and H4 answered question 2. In this process, the abilities of rural family business operators to run businesses played a moderating role, reinforcing the relationships between their business development cognition and financial awareness and the sustainable development of family businesses.

## Data Availability

The raw data supporting the conclusions of this article will be made available by the authors without undue reservation.
