# Supplementary material for: Financial awareness and business development cognition positively influence the sustainable development of rural family businesses
Source: Front Sociol. 2025 Jul 11;10:1569713. doi: 10.3389/fsoc.2025.1569713 (PMC12291685; doi:10.3389/fsoc.2025.1569713)
Supplement: Supplementary file 1 [file Table_1.docx]

Supplementary Material

# Supplementary Tables

Research instrument

| Constructs | Items | Measurements | Sources |
| --- | --- | --- | --- |
| Cognition of business development (E) | C1 | For sustainable development, I consciously evaluate to a very high extent alternatives with regard to customer problems and needs. | Schrauder et al., 2018 |
|  | C2 | For sustainable development, I consciously evaluate to a very high extent alternatives with regard to sales and distribution channels. |  |
|  | C3 | For sustainable development, I consciously evaluate to a very high extent alternatives with regard to business transactions and the ways of collaborating with partners. |  |
|  | C4 | For sustainable development, I consciously evaluate to a very high extent alternatives with regard to link business participants together in novel ways. |  |
|  | C5 | For sustainable development, I consciously evaluate to a very high extent alternatives with regard to take over new value chain positions or substitute existing parts of the value chain. |  |
| Financial awareness (I) | FA1 | I believe that the capital required for family business development should be based on own savings and should not be borrowed. | Liu et al., 2020 |
|  | FA2 | I believe that a bad credit history affects the financing loan process for family business development. |  |
|  | FA3 | I believe that the process of financial institutions for family business financing is moderate. |  |
|  | FA4 | I can attain loan for funds needed of family business development based on my family's risk tolerance. |  |
|  | FA5 | I believe that the term of borrowing for funds needed of family business development is the longer , the interest rate is the higher. |  |
| Abilities to run businesses (A) | A1 | One of my greatest strengths is achieving results by  organizing and motivating people. | Guo et al., 2013 |
|  | A2 | One of my greatest strengths is organizing resources and  coordinating tasks. |  |
|  | A3 | One of my greatest strengths is my ability to delegate effectively. |  |
|  | A4 | One of my greatest strengths is my ability to supervise,  influence, and lead people. |  |
|  | A5 | I make resource allocation decisions that achieve maximum  results with limited resources. |  |
| Sustainable development of family businesses (B) | B1 | I believe my business can continue to operate. | Zhao, 2023 |
|  | B2 | My business attracts relevant talent. |  |
|  | B3 | My company's target market is not yet saturated. |  |
|  | B4 | Our company has diverse sales channels. |  |
|  | B5 | Our company can provide products according to customer needs |  |
|  | B6 | I am satisfied with the profits of my business |  |
| Gender | | What is your gender? | |
| Education level | | What is your education level? | |
| Provinces | | What is your province? | |
